# Immunologic, metabolic and genetic impact of diabetes on tuberculosis susceptibility

**DOI:** 10.3389/fimmu.2023.1122255

**Published:** 2023-01-23

**Authors:** Phillip Ssekamatte, Obondo James Sande, Reinout van Crevel, Irene Andia Biraro

**Affiliations:** ^1^ Department of Immunology and Molecular Biology, School of Biomedical Sciences, College of Health Sciences, Makerere University, Kampala, Uganda; ^2^ Department of Internal Medicine and Radboud Centre for Infectious Diseases, Radboud University Medical Centre, Nijmegen, Netherlands; ^3^ Department of Internal Medicine, School of Medicine, College of Health Sciences, Makerere University, Kampala, Uganda

**Keywords:** tuberculosis, diabetes, immunity, metabolism, gene transcription

## Abstract

Due to the increasing prevalence of diabetes mellitus (DM) globally, the interaction between DM and major global diseases like tuberculosis (TB) is of great public health significance, with evidence of DM having about a three-fold risk for TB disease. TB defense may be impacted by diabetes-related effects on immunity, metabolism, and gene transcription. An update on the epidemiological aspects of DM and TB, and the recent trends in understanding the DM-associated immunologic, metabolic, and genetic mechanisms of susceptibility to TB will be discussed in this review. This review highlights gaps in the incomplete understanding of the mechanisms that may relate to TB susceptibility in type 2 DM (T2DM). Understanding these three main domains regarding mechanisms of TB susceptibility in T2DM patients can help us build practical treatment plans to lessen the combined burden of the diseases in rampant areas.

## 1 Introduction

### 1.1 Epidemiology and pathogenesis of diabetes mellitus

Diabetes mellitus describes a group of chronic metabolic disorders characterized and identified by the presence of hyperglycemia (1, 2). The diverse etiopathology includes defects in insulin secretion or action or both, and alterations in carbohydrate, lipid and protein metabolism (1). Globally, approximately 537 million adults (aged 20 to 79) were living with DM in 2021; a number that is expected to rise to 783 million by 2045 (2). Type 1 DM (T1DM) is attributable to the autoimmune destruction of the insulin-producing *β*-cells of islets of Langerhans by autoimmune antibodies, making patients insulin-dependent. Though, this doesn’t include the destruction of *β*-cells of islets of Langerhans for which specific causes are known such as cystic fibrosis (3, 4). This is a consequence of the formation of specific auto-islet *β-*antigens presented by antigen-presenting cells to activate islet antigen-reactive T-helper (Th1 and Th2) (5). Several studies have demonstrated the importance of islet-reactive *β* -cells in the pathogenesis of T1DM by presenting antigens to T cells and the production of cytokines and autoantibodies in mice and humans (6–8). Once activated, Th1 cells secrete interleukin (IL)-2 and interferon-gamma (IFN)-γ. IL-2 then activates cytotoxic T-cells to produce perforin and granzymes that destroy islet *β*-cells. IFN-γ activates macrophages to produce proinflammatory cytokines including IL-1β and tumor necrosis factor (TNF) which further destroy islet *β*-cells (9, 10).

**Figure 1:**
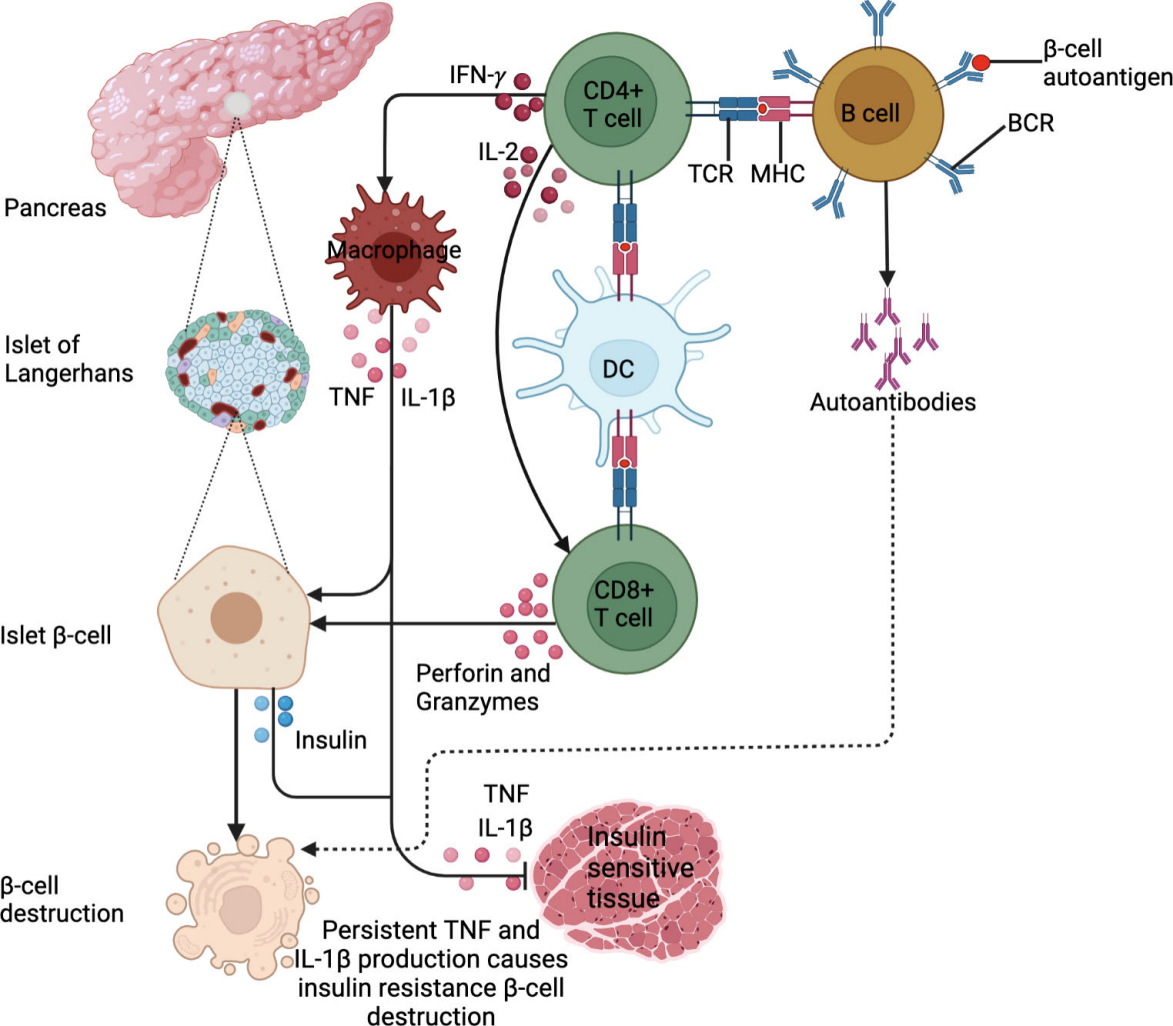
DM pathogenesis. DM is an immune-modulated disease. For T1DM, antigen presentation by B cells and DCs drives the activation of β−cell-specific T cells. In addition, the exposure of B cells to β−cell autoantigens leads to the production of islet β−cell targeting autoantibodies. These lead to β−cell destruction. For T2DM, IFN-*γ* production by activated CD4^+^ T cells activates macrophages to produce IL-1β and TNF. This low chronic-grade inflammation causes insulin resistance or inhibits insulin uptake in tissues. In addition, the cytokines cause insulin secretion by β-islet cells to compensate for reduced insulin sensitivity. These stimulate the Islet β cells to produce insulin. Persistent IL-1β and TNF production triggers β-cell islet destruction.

In contrast, T2DM is a chronic condition that occurs when body cells do not respond to insulin, renowned as “insulin resistance” thereby resulting in hyperglycemia (2). This state prompts a positive feedback cycle of insulin production, making the insulin ineffective over time (2). T2DM, the more prevalent diabetes subtype, accounts for approximately 90% to 95% of all diagnosed diabetes worldwide, with the highest proportions in low and middle-income countries (1). Understanding the direct causes of T2DM is not well elucidated, but there are strong links between overweight and obesity, advancing age, alcohol abuse, as well as ethnicity and a positive family history of DM. As with T1DM, T2DM results from a combination of multi-gene predisposition and environmental triggers (2). Obesity, specifically excessive visceral adiposity is associated with metabolic syndrome (hyperglycemia, dyslipidemia, insulin resistance and hypertension) (11). The progression from obesity-related insulin resistance to T2DM remains poorly understood, however, it involves a failure of pancreatic β-cells to compensate for insulin resistance resulting in chronic hyperglycemia. Abdominal obesity is associated with low-grade chronic inflammation and immune system activation, which may play a role in the aetiology of metabolic disorders linked to obesity, such as T2DM (12, 13).. White blood cell count (14), pro-inflammatory cytokines (TNF, IL-1β, IL-6) (15), chemokines including monocyte chemoattractant protein-1 (MCP-1), IL-8 and interferon-γ-inducible protein-10 (IP-10) (16), and several other indirect markers of inflammation including c-reactive protein (CRP), fibrinogen, sialic acid and plasminogen activator inhibitor 1 (PAI-1) (17), have been identified as predictors of T2DM. In obesity and T2DM, adipose tissue is characterized by an enrichment of macrophages and T-cells with a shift from an anti-inflammatory to a pro-inflammatory state (18, 19). Cytotoxic T-cells, Th1 and Th17 cells stimulate M1 macrophage polarization (18, 19). During obesity, an imbalance in T-cells, macrophages and other immune cells increases the production of chemokines and pro-inflammatory cytokines, which promotes systemic inflammation and insulin resistance (20). Subsequently, this immunological imbalance makes obese patients more susceptible to the development of T2DM, as shown in **Figure 1** below. The most common basis for diagnosing T2DM is by using either glycated hemoglobin (HbA1c) (≥ 6.5%), fasting glucose (126 mg/dl), random plasma glucose in patients with hyperglycemic symptoms (200 mg/dl), or a 2-hour plasma glucose after a 75g oral glucose tolerance test (200 mg/dl) (2). T2DM patients are prone to complications such as retinopathy, neuropathy, nephropathy, cardiovascular diseases and diabetic feet (2).

### 1.2 Epidemiology and pathogenesis of tuberculosis

**Figure 2:**
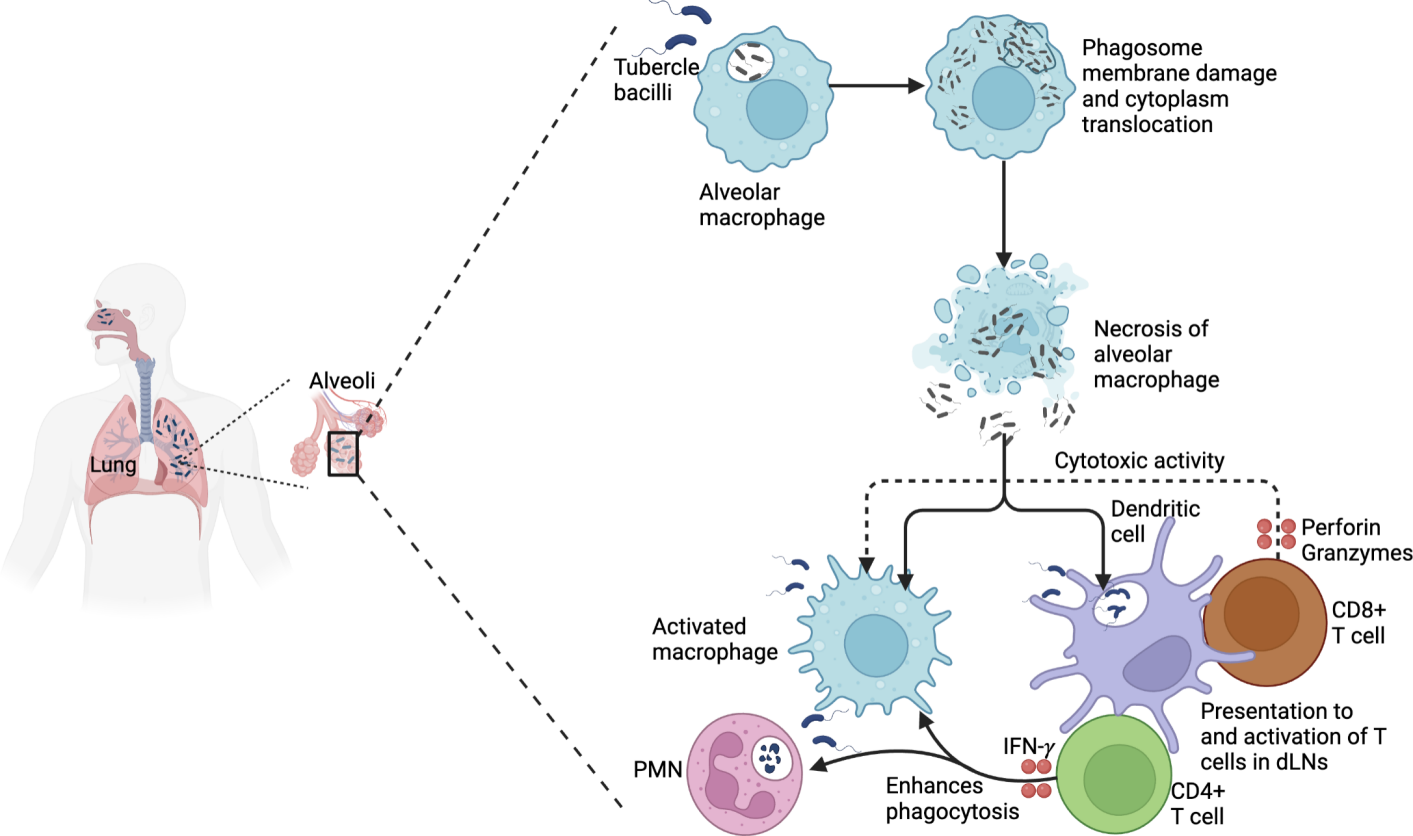
TB pathogenesis. Virulent *Mtb* that is phagocytosed by alveolar macrophages causes phagosome membrane damage and translocates to the cytoplasm. This results in necrosis and spread to other macrophages and dendritic cells. *Mtb* is processed and presented to CD4^+^ T cells that produce IFN-*γ*, enhancing the phagocytosis of infected macrophages and polymorphonuclear cells. In addition, activated CD8^+^ T cells produce perforin and granzymes that mediate the cytotoxic activity of infected macrophages.

According to the world health organization (WHO), in 2021 approximately 10.6 million people developed active TB (ATB) globally, of whom 1.4 million died (21). The prevalence of TB varies according to population. The majority of TB cases are found in South-East Asia (45%), Africa (23%), and the Western Pacific (18%) (21). TB was the leading cause of death from a single infectious agent and the 13^th^ largest cause overall in 2019 (21). Natural infection with *Mycobacterium tuberculosis* (*Mtb*) occurs by inhalation of aerosols infected with bacilli that are deposited on the primary alveolus (22). The tubercle bacilli then invade resident alveolar macrophages that provide the major initial replication niche for the pathogen (22). When viable tubercle bacilli are phagocytosed by alveolar macrophages, they secrete 6kDa early secretory antigenic target (ESAT-6). This peptide prevents phagosome-lysosome fusion and apoptosis, and promotes bacillus cytosolic translocation (23). So, the bacillus multiplies in a single alveolar macrophage, a process that develops over a week, making the alveolar macrophage necrotic (24). The tubercle bacilli then move extracellularly and are phagocytosed by alveolar macrophages from the interstitial space and those of neighboring alveoli. Continuous repetition of this process generates enough tubercle bacilli to stimulate infected alveolar macrophages to produce an inflammatory response (24). Polymorphonuclear (PMN) cells and monocytes enter the alveoli, leading to more vigorous phagocytosis in the affected alveoli, and drainage into lymph nodes. This infects and generates myeloid dendritic cells (mDCs) (24). However, it is to be noted that mDCs also convey tubercle bacilli to lymph nodes once infected (25). These dendritic cells process *Mtb* and present epitopes that mostly correspond to the most abundant antigens secreted including ESAT-6 and the antigen 85 complex (Ag85 A, B or C) (26). The antigen presentation stimulates the CD4^+^ T-cell proliferation and differentiation into subsets, including Th1, Th17 and regulatory T cells (Tregs). CD8^+^ T cells may also be stimulated and proliferate on a small scale (27). These T-cells traffic to the lung, where IFN-γ is produced from the Th1-cells to stimulate macrophage antimycobacterial specific cytokine production and cytotoxicity targeting of *Mtb* infected macrophages (28). This T-cell-mediated activation of macrophages and other immune cells including neutrophils, NK cells, B cells, and DCs restricts *Mtb* replication through formation of granuloma (29). Primary ATB may develop in case the immune system and the granuloma cannot control the initial spread of *Mtb* infection, especially in immune-compromised persons (30). If the immune system and granuloma contain *Mtb* but do not eliminate the bacteria, the person has latent TB infection (LTBI), which can progress to ATB at a later stage (30). Immunosuppression can lead to reactivation of *Mtb* within the granuloma, resulting in pulmonary TB, extrapulmonary TB or miliary TB. Pulmonary TB, the most common form of TB, is characterized by cough, fever, anorexia, weight loss, night sweats and chest X-ray abnormalities (30). Miliary TB involves the hematogenous spread of granuloma throughout the body, while extrapulmonary TB involves lymph nodes, bones, gastrointestinal and other organ systems (30). LTBI is characterized by immunoreactivity to *Mtb*, in the absence of clinical symptoms or radiological abnormalities suggestive of ATB. Immunological memory to *Mtb* is measured by the tuberculin skin test (TST) or an IFN-γ release assay (IGRA). The lifetime risk for reactivation of LTBI is 5% to 15% (31). The distinction between LTBI and ATB can be subtle, as an estimated 50% of patients with culture-positive ATB are asymptomatic (‘subclinical TB’) (32), even though they can transmit the tubercle bacilli to others (33). For the detection of ATB, three techniques are employed: microbiological tests (microscopy, culture, molecular tests), imaging and histopathological examination. Whereas imaging techniques are employed in screening, microbiological analysis is required for ATB diagnosis. This is because X-rays have low specificity and therefore abnormal chest X-rays are followed up with microbiological tests (34). It is to be noted however that the recent emergence of digital radiology and computer-aided diagnostic software is providing new insights into the diversity of lung lesions (35). Owing to its superior sensitivity and specificity to sputum smear microscopy, the WHO now recommends Xpert MTB/RIF as the first-line diagnostic test in all adults or children who are suspected of having ATB (36). **Figure 2** summarizes the TB pathogenesis.

## 2 Epidemiological effects of T2DM on TB

### 2.1 T2DM increases the risk of LTBI and ATB

T2DM has been reported as a primary *Mtb* infection risk factor (37, 38). Patients with T2DM (43.4%) and pre-diabetes (39.1%) had significantly more LTBI than those without T2DM (25.9%), with a strong association between T2DM and LTBI (adjusted odds ratio: 2.3, 95% confidence interval: 1.2-4.5) (39). In addition, another study reported T2DM to be strongly associated with increased LTBI risk (pooled odds ratio: 1.18, 95% confidence interval: 1.06–1.30) (40). A large cross-sectional study of the US national data also reported that T2DM significantly increases LTBI risk (adjusted odds ratio: 1.90, 95% confidence interval: 1.15–3.14) (37). Correspondingly, a systematic review involving 20 studies also reported a significant LTBI risk by T2DM (for cohort studies, relative risk: 1.62, 95% confidence interval: 1.02–2.56; for cross-sectional studies, odds ratio: 1.55, 95% confidence interval: 1.30–1.84) (38). The association between T2DM and ATB has been well established (41), with T2DM increasing the risk for ATB development by greater than 3-fold (42). T2DM predisposes individuals to the acquisition of LTBI and the development of ATB, with this expected to escalate with the increasing T2DM prevalence (2).

### 2.2 T2DM increases the risk of multi-drug-resistant TB

T2DM has been linked with an increased multi-drug resistant TB (MDR-TB) risk. A meta-analysis reported significantly increased rates of MDR-TB in T2DM patients (odds ratio: 1.97, 95% confidence interval: 1.58–2.45) (43). In addition, another systematic review and meta-analysis reported an approximately 2-fold MDR-TB increased risk in T2DM patients (odds ratio: 1.97, 95% confidence interval: 1.58–2.45) (44). Using whole genome sequencing, T2DM was associated with mutations conferring resistance to isoniazid and ethionamide (Rv1482c-fabG1) and fluoroquinolone (gyrA) in *Mtb* isolates of ATB patients, with the association evident even among patients with newly diagnosed TB (45).

### 2.3 T2DM increases TB disease severity, treatment failure and relapse

Upon chest X-ray, cavities and infiltration have been reported in ATB patients with T2DM (46), implying that the patients have more severe disease. In addition, CT scans revealed bilateral pulmonary involvement and extensive pulmonary disease in TB patients with T2DM (47). In addition, poorly controlled T2DM was associated with all-lobe involvement, advanced extensive lesions and more cavities (47). T2DM is significantly associated with adverse TB treatment outcomes and mortality (42, 44). In addition, T2DM is associated with early mortality during TB treatment (adjusted hazard ratio: 4.36; 95% confidence interval: 1.62–11.76) (48), and more than a 2- fold increased risk of death (2.16 times) (49).

## 3 Effects of T2DM on TB functional immune responses

### 3.1 Effects of T2DM on TB innate immune responses

Innate immune cells primarily consist of macrophages, innate lymphoid cells (ILCs), neutrophils and DCs. They roam in alveolar tissue and blood where they identify *Mtb* using pattern recognition receptors (PRRs), initiating a series of immune mechanisms including autophagy, apoptosis, and phagocytosis that kill the *Mtb* (50). The function of these innate immune cells may be compromised by metabolic dysregulation in T2DM.

Macrophages play a central role in the control of *Mtb* through the production of antimicrobial agents including reactive nitrogen and oxygen species, and cytokines. Other macrophage types such as monocyte-derived macrophages (MDM) are recruited to sites of infection (50). The expression levels of HLA-DR on H37Rv-infected MDMs of T2DM patients are decreased, while those of PD-L1 are increased (51). PD-L1 inhibits T cell proliferation, cytokine production and cytolytic function. Upregulated PD-L1 expression inhibits the Th1 immune response of the macrophages resulting in *Mtb-*mediated macrophage susceptibility. In addition, alveolar macrophages from diabetic mice infected with *Mtb* have increased CCR2 expression, which decreases monocyte homing to the lungs. Furthermore, these macrophages exhibit reduced expression of CD14 and macrophage receptor with collagenous structure (MARCO). These function in recognition of the bacterial cell wall component trehalose 6,6′-dimycolate (TDM) (52), promoting the susceptibility of diabetic hosts to TB. In addition, oxidized low-density lipoprotein (oxLDL)-derived free and esterified cholesterol sequestered within lysosomes are reported to induce lysosomal dysfunction, supporting the survival of *Mtb* within macrophages in TB-T2DM comorbid patients (53). These data show that T2DM alters the macrophage activation and function state, impacting the ability of macrophages to eliminate *Mtb* in patients with TB-T2DM.

Neutrophils accumulate and peak within 24 hours after *Mtb* infection or BCG vaccination in murine and rabbit models (54) and have shown protection in early tuberculous granuloma in a zebrafish model by oxidatively killing mycobacteria inside macrophages (55). In humans, neutrophils are an abundant *Mtb*-infected cell type early in infection, within which *Mtb* rapidly replicates (56). Patients with ATB-T2DM have elevated levels of absolute neutrophil counts, but these have decreased adhesion abilities and result in impaired phagocytosis of *Mtb* (57). Thus, T2DM reduces neutrophil antibacterial activity consequently increasing *Mtb* risk in T2DM patients. Heightened neutrophil sub-sets have been linked to inflammation (58, 59) and TB severity, and recovery pre-and post-treatment (59). Moreover, Berry et al. used modular and pathway analysis to reveal a whole blood neutrophil-driven interferon (IFN)-inducible gene profile that consisted of both IFN-γ and type I IFNαβ signaling correlating with ATB (60). Prada-Medina et al. demonstrated that this correlation is exacerbated in ATB-T2DM patients, with neutrophils as the inflammatory nexus between TB and T2DM (61). The systemic chronic low-grade inflammation, a characteristic of T2DM, impairs TB immune responses (62). IL-8 levels are elevated in patients with TB-T2DM compared to TB-only and healthy controls, and these are strongly positively associated with proinflammatory cytokines including TNF and IL-6 (63), and neutrophil recruitment during *Mtb* (64). Resistin, a soluble serum protein produced majorly by neutrophils (65), causes insulin resistance and mediates the progression from obesity to T2DM (66). Elevated resistin levels have been reported to impair chemotaxis and reactive oxygen species (ROS) production by neutrophils in ATB-T2DM patients (67). Mechanistically, exogenous resistin is reported to inhibit ROS and IL-1β production by macrophages, suppressing the inflammasome, and resulting in exponential *Mtb* growth (67). This shows that neutrophils may be central to the TB-T2DM pathology for targeted host-directed therapies.

Dendritic cells (DCs) are the most professional antigen-presenting cells (APCs), activating naïve T cells to initiate adaptive immune responses (68). Hence, the killing of *Mtb-*infected cells is also heavily dependent on the T cell-mediated immune responses. Kumar et al. reported impaired myeloid and plasmacytoid DC frequencies in patients with ATB-T2DM at baseline and 2 months of anti-TB treatment, and the DC frequencies were reversed at 6 months of anti-TB treatment (69). Reduced DC frequencies impair the ability of DCs to prime T cells which impacts the host’s ability to kill and clear *Mtb*. Mechanistic studies, however, need to evaluate the function of DCs in TB-T2DM comorbidity.

Innate lymphoid cells (ILCs) are tissue-resident cells especially found in the intestine (70), lungs (71) and skin (72), with an ability to quickly respond to pathogens. Tripathi et al. evaluated the protective role of ILC3 and IL-22 in regulating mortality and inflammation in *Mtb*-infected diabetic mice. IL-22 produced by ILC3 was lower in *Mtb*-infected diabetic mice compared to controls. Recombinant IL-22 treatment and ILC3 adoptive transfer improved lipid metabolism and prolonged *Mtb*-infected diabetic mice survival (73). In humans, we reported lower IL-22 production by ILC3 (74), and that IL-22 production by ILC3s was critical for early innate immunity and granuloma formation (75). T2DM probably inhibits IL-22 production, and this pathway may be a potential host-targeted therapy for intervention in TB-T2DM comorbidity. Recent nomenclature has grouped NK cells as ILC1 (76). NK cell frequencies are reported to be elevated, with CD16 and CD56 levels being highly expressed in ATB-T2DM patients (77). Interestingly, CD16 and CD56 expression levels decreased following anti-TB treatment (77), highlighting the clinical significance of the NK cells in the treatment monitoring of TB-T2DM patients. NK cells and CD11c are reported to interact producing IL-6 that inhibits CD4^+^ T cell proliferation. This reduces the Th1 and Th17 cellular immune responses, exposing diabetic mice to *Mtb* and reducing the survival rate of the mice (78).

### 3.2 Effects of T2DM on TB adaptive immune responses

**Figure 3:**
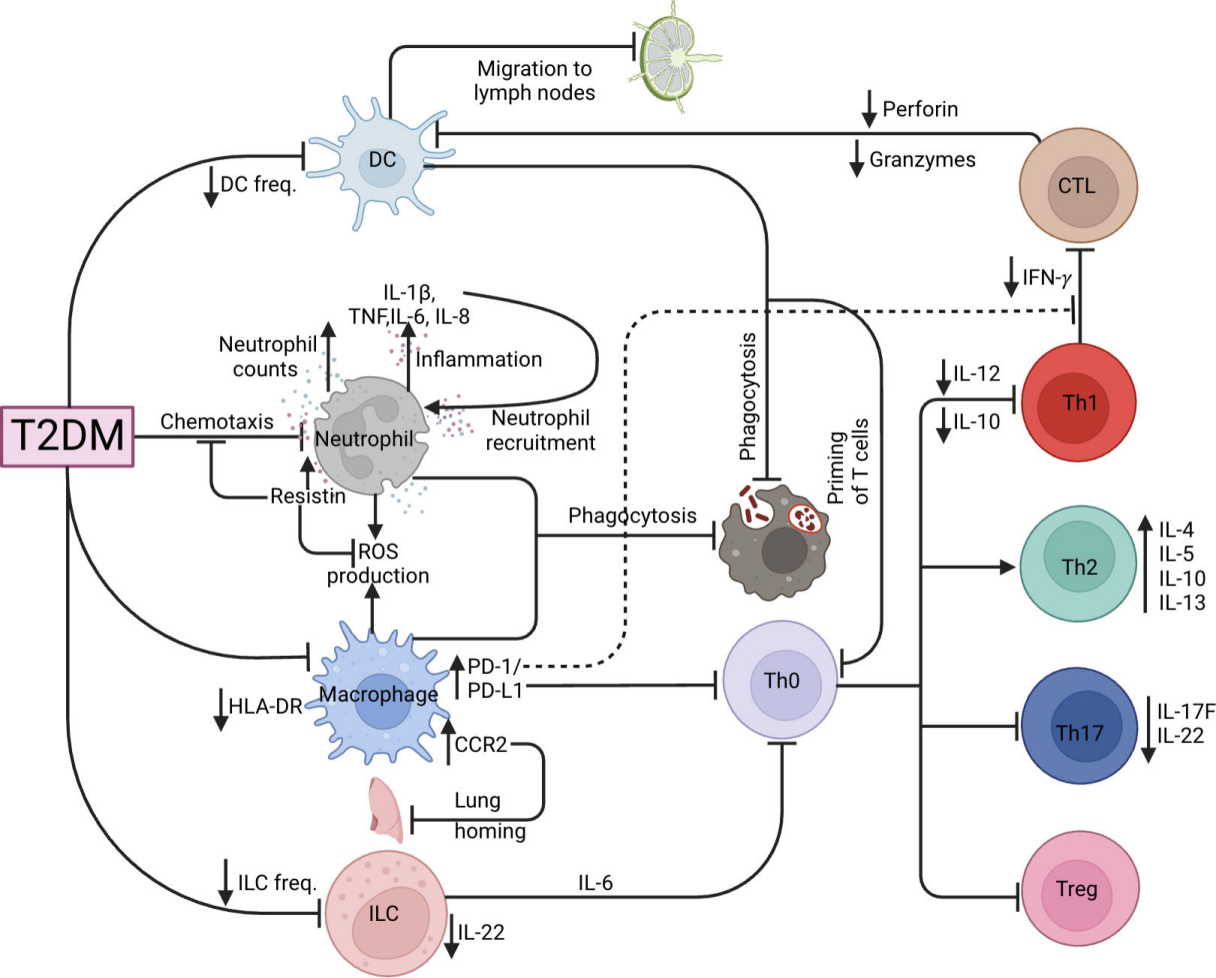
TB and T2DM immunological dysregulation. Effects of T2DM on the various innate (DC, neutrophil, macrophage, ILC) and adaptive (CTL, Th1, Th2, Th17 and Tregs) immune cells.

T-helper type 1 (Th1), Th17 responses and the balance of the Th1/Th2 ratio are necessary for controlling *Mtb* pathogenesis (79, 80). It has been reported that Th2 and Th17 cells are significantly increased while Th1 cells were unchanged in ATB/T2DM patients, decreasing the Th1/Th2 ratio, with a lower proportion of CD8^+^ cytotoxic T cells (81). Moreover, ATB-T2DM patients are characterized by elevated frequencies of CD4^+^ Th1 and Th17 cells, but lower frequencies of regulatory T (Treg) cells as compared to ATB patients without T2DM (82). In addition, LTBI-T2DM patients have reduced CD4^+^ Th1, Th2, and Th17 cells (83). Correspondingly, Faurholt-Jepsen et al. (84) reported that T2DM is associated with diminished *Mtb* antigen-specific IFN-γ production in ATB patients. Interestingly, IL-12, a potent promoter of IFN-γ production, is reported to be lowered in ATB-T2DM patients and impairs the ability of Th1 cells to produce sufficient IFN-γ levels to control *Mtb* infection (85). The authors further reported heightened IL-10 levels that were associated with Th1 response inhibition in ATB/DM compared to ATB alone patients (85). Other immune parameters have been associated with a decreased TB-specific Th1 response. Lopez-Lopez et al. demonstrated that *Mtb*-infected MDMs of T2DM patients had increased expression of PD-L1 (51), and the PD-1/PD-L1 pathway inhibited the Th1 response and consequently decreased IFN- γ production (86). Taken together, the Th1 and Th2 imbalance as a result of diminished Th1 responses during T2DM impairs the ability of the host to eliminate TB. T cells including their memory subsets such as central memory and effector memory T cells have been shown to play critical roles in protective immune responses in animal models of vaccination (87). ATB-T2DM patients had elevated frequencies of central memory CD4^+^ and CD8^+^ T cells and decreased frequencies of naïve, effector memory, and/or effector CD4^+^ and CD8^+^ T cells at baseline and after two months of treatment, but not after six months of treatment in comparison to ATB-T2DM patients (88). This shows that CD4^+^ and CD8^+^ T cells and their memory subset are restored after anti-TB treatment. Hence collectively, this data shows that T2DM has profound effects on CD4^+^ and CD8^+^ T cells and their memory subset but is restored following anti-TB treatment. More studies need to assess the functional responses among these memory T cell subsets as well as alternations in the CD8^+^ T cell cytotoxicity need to be further studied during anti-TB-treatment. During LTBI, T2DM patients have diminished type 1 (TNF, IL-2 and IFN-γ), type 17 (IL-17F), pro-inflammatory (IL-1 and IL-18) cytokines, as well as the anti-inflammatory cytokine IL-10 (89). This may be attributable to T2DM potentially directly influencing and/or decreasing the frequency of Th1 cells in LTBI-T2DM patients, suggesting that T2DM is associated with a general decrease of CD4^+^ T cell subset and cytokine responses. Following *Mtb*-antigen stimulation, patients with LTBI-T2DM showed lower frequencies of CD8^+^ Tc1, Tc2, and Tc17 cells, with elevated cytotoxic markers (perforin and granzymes) than those without T2DM and reversing upon ATB development (90). In conclusion, T2DM compromises the immunological responses to *Mtb*, resulting in the underproduction of protective CD4^+^ and CD8^+^ T-cell responses, potentially increasing ATB susceptibility, as summarized by **Figure 3**.

## 4 Effects of T2DM on the immunometabolic and gene-transcriptional mechanisms for TB susceptibility

### 4.1 Effects of T2DM on the immunometabolic mechanisms of TB susceptibility

**Figure 4:**
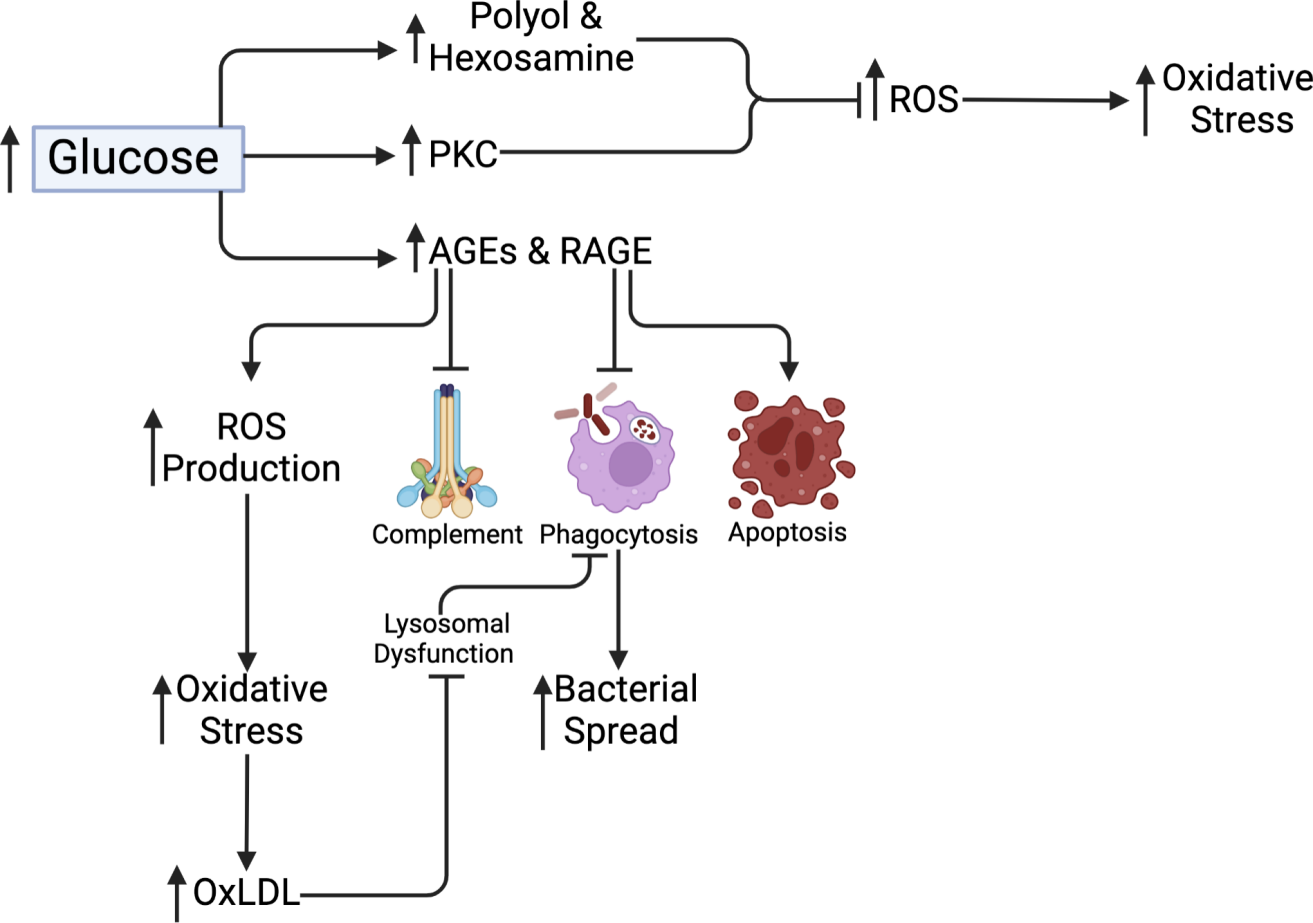
Metabolic pathways related to hyperglycemia. Polyol and Hexosamine and PKC pathways cause increased ROS production and uptake resulting in increased mitochondrial stress. AGEs and RAGE production inhibits complement activation and macrophage phagocytosis, as well as promote macrophage apoptosis and increased ROS production. This increases oxidative stress, resulting in increased OxLDL. OxLDL causes lysosomal dysfunction and phagocytosis, which promote bacterial spread.

The major upstream event triggering complications in T2DM, according to Brownlee and Giacco (91, 92), is hyperglycemia-dependent mitochondrial superoxide overproduction. These pathways include increased polyol and hexosamine flux; increased protein kinase C (PKC) isoform activation; increased advanced glycation end-products (AGEs) formation; and increased receptor for advanced glycation end-products (RAGE) and the expression of its endogenous ligand. In general, all these pathways induce oxidative stress by upregulating ROS production, increasing pro-inflammatory signaling and cellular and tissue changes and damage (92). In poorly controlled DM, highly glycated proteins and AGEs are prevalent. Glycation of proteins and the production of AGEs impede complement activation, bacterial absorption *via* phagocytosis, and phagocytic killing, resulting in mycobacterial spread (93). After *Mtb* infection, certain AGEs intermediates, including methylglyoxal, induce macrophage apoptosis (94). The stimulation of the mitogen−activated protein kinase (MAPK) pathway or the production of NF-kB as a result of RAGE interaction activates the NLRP3 inflammasome inducing IL‐1β and IL-18 secretion leading to inflammation, which is one of the suggested mechanisms for AGE action (95). In addition, AGE induces excessive mitochondrial ROS production causing oxidative stress and impaired wound healing (96). Similar to AGE, oxidized low-density lipoprotein (oxLDL) is a pathologically altered lipoprotein that is increased due to oxidative stress in T2DM patients (97). To explore this, Palanisamy et al. found that guinea pigs infected with *Mtb* had associated increased macrophage scavenger receptor expression and oxLDL accumulation in granulomas supported intracellular bacilli survival and persistence (98). Another study in humans reported oxLDL supporting *Mtb* survival in macrophages by inducing lysosomal dysfunction (53). The authors reported improved macrophage lysosomal function following anti-oxLDL treatment. Correspondingly, metformin treatment has anti-mycobacterial benefits on cellular metabolism, immune function, and gene expression. In mice and T2DM patients, metformin-educated CD8^+^ T cells had increased oxidative phosphorylation, survival capacity and anti-mycobacterial properties (99). Correspondingly, metformin upregulates genes involved in ROS production and phagocytosis, while downregulating type 1 IFN response genes and inflammation (TNF-α, IL-1β, IL-6, IFN-γ, and IL-17) following *Mtb* stimulation (100). **Figure 4** summarizes the metabolic pathways related to hyperglycemia.

T2DM and obesity are linked to gut microbiome dysbiosis, which results in changes in the species that produce short-chain fatty acids (SCFAs) (101). SCFAs are bacterial metabolites that alter the activity of different cell types, including lymphocytes, neutrophils, and macrophages, hence modulating inflammatory and immunological responses (102). SCFAs have been reported to regulate glucose homeostasis mechanisms (103). In addition, intestinal microbiota has been reported to regulate LPS levels, which may promote diabetes development (104). Compared to normal glucose tolerance individuals, T2DM patients have an elevated abundance of four *Lactobacillus* species and a decreased abundance of five *Clostridium* species. In addition, *Lactobacillus* species positively correlated with fasting glucose and HbA1c, while *Clostridium* species are negatively correlated with HbA1c, fasting glucose and plasma triglycerides (105), linking the *Lactobacillus* taxa to the development of T2DM. Several studies have reported changes in the gut microbiota of TB patients compared to healthy controls. Infection with *Mtb* decreased α diversity, specifically with changes in *Bacteroides* relative abundance (106). SCFA-producing bacteria were decreased in TB patients compared to controls (107), while Firmicutes and Actinobacteria were increased in TB patients (108). Using a diabetic rat model, Sathkumara et al. reported altered gut microbiota in diabetic mice, and the microbial diversity was further decreased in diabetic mice that were *Mtb* aerosolized (109). Butyrate, an SCFA modulates mucosal immune responses suppressing the activation, differentiation and recruitment of neutrophils, macrophages and DCs (110). *Mtb* aerosolized diabetic mice show an abundance of butyrate‐producing *Firmicutes* (109). Butyrate treatment has been associated with decreased production of *Mtb*‐induced pro‐inflammatory cytokines such as IL‐1β, TNF‐α and IL‐17A, and increased IL‐10 production (111). In addition, butyrate inhibits the activation of antigen-specific CD8^+^ T cells and reduced the secretion of IL-12p70 and expression of costimulatory molecules including CD80/CD83/CD40 and MHC-I/II by DCs (112). This demonstrates that altered microbiota influences TB immune responses including markers of antigen recognition and presentation, making T2DM patients susceptible to TB. **Table 1** summarizes the influence of gut microbiota in the context of TB and T2DM.

**Table 1:** Influence of gut microbiota in the TB and T2DM context.

Study	Microbiota differences	Importance
**T2DM** (105)	↑*Lactobacillus gasseri* JV-V03, *Lactobacillus gasseri* SJ-9E-US, *Lactobacillus gasser*i 202-4, *Lactobacillus salivarius* ACS-116-V-Col5a ↓*Clostridium beijerinckii* NCIMB 8052, *Clostridium* sp. 7_2_43FAA, *Clostridium botulinum* B str. Eklund 17B, *Clostridium botulinum* E3 str. Alaska E43, *Clostridium thermocellum* DSM 1313	Development of T2DM
**LTBI and ATB** (106–108)	↑*Firmicutes* and Actinobacteria ↓Alpha diversity and SCFA-producing bacteria	Promotion of TB susceptibility
**ATB and T2DM** (109)	↑*Firmicutes*, Lactobacillaceae, Erysipelotrichaceae ↓Bacteroidetes, Muribaculaceae, Akkermansiaceae, Ruminococcaceae, Lachnospiraceae	Promotion of TB susceptibility

**Figure 5:**
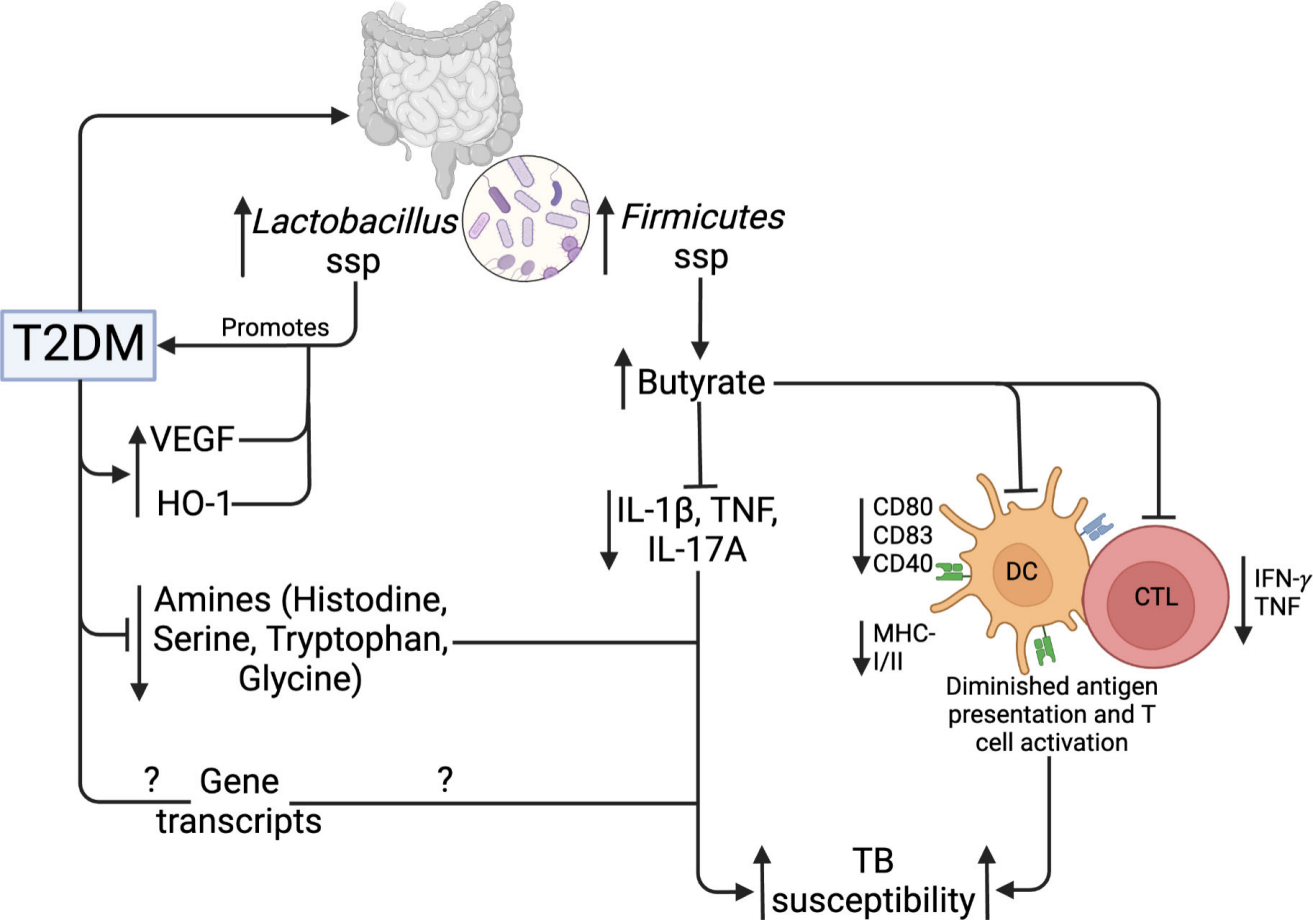
Gut microbiome, metabolic and genetic dysregulation in T2DM and TB. T2DM upregulates *Lactobacillus* and *Firmicutes* taxa of the gut microbiota. *Lactobacillus* promotes further T2DM development, while *Firmicutes* upregulate butyrate production. Butyrate inhibits *Mtb*-induced cytokine production, activation, and antigen presentation by DCs and CTLs. In addition, T2DM lowers specific amino acids and gene transcripts. Collectively, these mechanisms increase TB susceptibility.

An alternate method that can dissect the pathophysiology of ATB-T2DM comorbidity is metabolomics, giving way to full-scale analysis for the study of biomarkers and how they are important in the prediction of disease. Biomarkers may include cytokines, mycobacterial antigens, metabolic activity markers and volatile organic compounds (113). Andrade et al. reported that patients with ATB-T2DM had elevated plasma levels of haem oxygenase-1 (HO-1) than those with ATB, and the levels positively correlated with random plasma glucose, LDL levels and HbA1c (114). ATB-T2DM comorbidity is characterized by elevated circulating levels of inflammatory cytokines and vascular endothelial growth factors (VEGFs), and the levels are positively correlated with HbA1c levels (115). Taken together, plasma levels of HO-1 and VEGF could be potential biomarkers of pathogenesis in TB with T2DM. More studies have further reported specific plasma metabolites in ATB-T2DM using high-throughput metabolomics techniques. A study by Vrieling et al. used targeted tandem liquid chromatography-mass spectrometry (LC-MS/MS) to compare amine levels in plasma samples of patients with ATB or ATB-T2DM, and reported that amine levels (citrulline, histidine, ornithine, tryptophan, serine, homoserine, glycine and threonine) were strongly decreased in ATB-T2DM group compared ATB to healthy control groups (116). The diverging amine metabolites were restored to healthy levels following antibiotic treatment. In addition, Choline, serine and putrescine biomarkers showed the highest potential for discriminating ATB-DM from TB patients (116). Moreover, in addition to altered metabolites, it is reported that Phenylalanine/Histidine metabolite ratio had a high predictive capacity as a biomarker for TB regardless of DM status (116, 117). Similarly, an increased Kynurenine/Tryptophan metabolite ratio is reported and correlates with enhanced activity of Indoleamine 2,3-dioxygenase (IDO), an immunoregulatory enzyme (116). The Kynurenine/Tryptophan metabolite ratio benefits *Mtb* infection and showed potential as a biomarker for TB diagnosis (118). These findings demonstrate the value of better blood metabolite and lipid control in the treatment of ATB-T2DM. These studies, taken together, significantly improve our understanding of metabolic changes in coincident ATB-T2DM and identify novel biomarkers for the diagnosis and prognosis of TB, shown in **Figure 5**.

### 4.2 Effects of T2DM on the gene-transcriptional mechanisms of TB susceptibility

The blood transcriptome provides insight into immunological events in the lungs as well as a gene expression signature for ATB and T2DM. Using host blood transcriptomic biomarkers, rapid point-of-care screening, diagnostic, and predictive tests for ATB could be developed. Several studies have been done to understand the gene transcriptional mechanisms of ATB susceptibility in people with T2DM. A study by Prada et al. reported considerable heterogeneity in differentially expressed genes (DEGs) with more DEGs identified in the ATB-T2DM or ATB groups than in the T2DM and healthy groups (61). These were linked to elevated circulating plasma cytokines and growth factors, reporting that the immune response to *Mtb* infection is the primary driver of ATB-T2DM patients’ blood transcriptomic changes (61). Interestingly, another study reported an increase in genes associated with innate inflammatory responses and neutrophils and a decrease in adaptive immune responses (119). Diagnostic signatures that distinguish ATB from LTBI and other disease states have been found using systems biology techniques in the past. In this regard, a 393-gene ATB signature was identified and validated by Berry et al. allowing ATB and LTBI to be distinguished (60). In addition, a TB-specific 86-whole blood signature was identified that discriminated TB from other bacterial and inflammatory diseases, with a pooled specificity of 83% (60). Anderson et al. identified and validated a 51-gene profile that differentiated ATB from other diseases in African children with and without HIV infection, with a sensitivity of 82.9% and a specificity of 83.6% (120). Other studies have identified RNA blood signatures that predict the risk of progression to ATB. A prospective blood RNA signature for predicting TB risk (121) identified and validated a 16-gene signature of risk that predicted tuberculosis progression in the 12 months preceding tuberculosis diagnosis. The signature predicted tuberculosis progression with a sensitivity of 66·1% and a specificity of 80·6% (121). The signature was reduced to 11 genes by *Scriba et al.* (122). The RISK11 signature distinguished between patients with prevalent tuberculosis or progression to incident tuberculosis, patients who remained healthy, and patients with at least one symptom consistent with TB had RISK11 scores of more than 80% (122). In addition, the provision of a once-weekly isoniazid-rifapentine for 12 weeks (3HP) regimen to signature-positive individuals after the exclusion of baseline disease did not reduce the progression to tuberculosis over 15 months (122). Adam et al. identified and validated a 6-gene transcriptomic signature profile for identifying persons at risk of incident TB, for subclinical TB triage and TB treatment monitoring (123). Moreover, the ROC curve exceeded 85% for subclinical and clinical disease diagnosis, and a sensitivity of 90% met the benchmarks set out in World Health Organisation target product profiles (TPP) for non-sputum/blood-based tests (123). In the TB-T2DM context, a recent study has reported two blood transcriptional signatures (8 and 22 signatures) that distinguished patients with poor and good TB treatment outcomes irrespective of T2DM condition (124). Interestingly, an overlap of TB risk signature genes (*GBP1, GBP2, GBP5, FCGR1A, STAT1, TAP1*) that predicted TB development from healthy controls from previous studies was reported (124). Though extensive host-blood transcriptomic biomarker research has been done in TB/HIV, little to no studies have assessed the risk and the performance of these biomarkers in the case of TB-T2DM comorbidity. Taken together, the diverse platforms used for blood-based transcriptomic biomarker signatures highlight the robustness of the methods used and the resulting data, as well as the potential for TB prediction and diagnosis. They also provide insights into further research on the risk of ATB in patients with T2DM. **Table 2** summarizes the list of genes associated with TB disease risk, and in the context of T2DM.

**Table 2:** Genes associated with TB disease risk, and in the context of T2DM.

Signature	Genes differentially expressed	Sensitivity	Specificity	Reference
**Berry393**	↑ OAS1, IFI6, IFI44, IFI44L, OAS3, IRF7, IFIH1, IFI16, IFIT3, IFIT2, OAS2, IFITM3, IFITM1, GBP1, GBP5, STAT1, GBP2, TAP1, STAT1, STAT2, IFI35, TAP2, CD274, SOCS1, CXCL10, IFIT5 ↓ All other genes	90%	83%	(60)
**Anderson51**	↑ACTA2, APOL6, CARD16, CLIP1, DEFA1, DEFA1B, DEFA3, GBP5, GBP6, LOC400759, RAP1A ↓ALKBH7, C11ORF2, C20ORF201, C21ORF57, C8ORF55, CRIP2, DGCR6, DNAJC30, E4F1, FBLN5, GNG3, HS.538100, IMPDH2, KLHL28, LCMT1, LGTN, LOC389816, LRRN3, MFGE8, NDRG2, NME3, NOG, PAQR7, PASK, PHF17, SIVA, SNHG7, TGIF1, U2AF1L4, UBA52	82.9%	83.6%	(120)
**Zak16**	↑ANKRD22, APOL1, BATF2, ETV7, FCGR1A, FCGR1B, GBP1, GBP2, GBP4, GBP5, SCARF1, SEPT4, SERPING1, STAT1, TAP1, TRAFD1	66.1%	80.6%	(121)
**RISK11**	↑STAT1, GBP2, GBP1, SERPING1, SCARF1, ETV7, TAP1, BATF2, GBP5, TRAFD1, FCGR1C	35%	91%	(122)
**RISK6**	↑GBP2, FCGR1B, SERPING1 ↓TUBGCP6, TRMT2A, SDR39U1	90%	55.7%	(123)
**Cassandra8&22**	↑GNLY, PRF1, CD3E, PTPRCv1, NLRP1, BCL2, CCR7, TAGAP, IFIT5, CXCL19 ↓GBP1, GBP2, GBP5, IFITM3, STAT2, MMP9, IRF7, IFI6, IFIT2, IFIT3, TAP2	Not reported	Not reported	(124)

## 5 Current challenges and future perspectives

### 5.1 The TB-DM pathogenesis remains unclear

Several studies have reported that T2DM has profound effects on TB treatment outcomes, and impairs the immune, metabolic and gene transcriptional mechanisms in response to *Mtb* infection, which may promote the development of ATB. These studies report highly heterogenous results making it impossible to point to one mechanism as the main cause of TB susceptibility in T2DM. In addition, TB has several stages including LTBI, incipient TB, subclinical TB, and ATB, making the gap in knowledge of these stages in the context of T2DM even wider. Correspondingly, the clinical presentation and prognosis of T2DM show considerable heterogeneity, with the clustering of 32 phenotypes identifying 4 archetypes with different dysfunctional patterns across T2DM etiological processes (125). Moreover, in the context of Africa, there is a relative scarcity of epidemiological data compared to Asia, with a 2.77 TB disease risk previously reported (126). The influence of HIV and COVID-19, major risk factors in the TB-T2DM comorbidity need to be assessed. Therefore, the lack of enough knowledge about the pathogenic mechanisms of TB in the context of T2DM accounts for the lack of unified standards in experimental design that would elucidate the exact mechanisms, which consequently leads to the design of better predictive prognostic markers and tests and treatment options.

### 5.2 The emergence of new technologies and platforms will help dissect the mechanisms of TB-T2DM pathogenesis, and the prediction and prognosis of TB

Advances in scientific research have led to a shift from technologies that assess dysregulation or impairment using the genome or proteome to platforms that provide a comprehensive analysis of metabolites and genes that could be dysregulated. This provides further insights into the mechanisms of impairment that could decipher the TB-T2DM pathogenesis. Omics which includes proteomics, transcriptomics, and metabolomics provide an enhanced understanding of molecular and metabolite dysregulation. This further enhances studies that research blood-based biomarkers of metabolism, immunity and transcriptome that predict and diagnose patients at high risk of developing ATB. This relieves the pressure on the healthcare systems.

## 6 Conclusion

T2DM has a considerable negative impact on public health by increasing the risk and severity of ATB by a 3-fold and worsening TB treatment outcomes, though a lower risk ratio has been reported in sub-Saharan Africa. Given the complex array of mechanisms and pathways involved in T2DM and TB pathology, the actual mechanisms that underpin TB susceptibility under T2DM are not well elucidated. A better understanding of the immunologic, metabolic, and genetic mechanisms for TB susceptibility in T2DM would contribute to rationally devising practical treatment methods to reduce the dual burden of both diseases. Because these T2DM complex mechanisms will likely affect TB immune responses, critically assessing perturbation of metabolic and genetic pathways caused by T2DM will undercover alterations in rather protective immune responses against TB.

## Author contributions

PS, OJS, RvC, and IAB conceptualized the study. PS searched the literature and drafted the manuscript. All authors contributed to the article and approved the submitted version.
